# Recovirus NS1-2 Has Viroporin Activity That Induces Aberrant Cellular Calcium Signaling To Facilitate Virus Replication

**DOI:** 10.1128/mSphere.00506-19

**Published:** 2019-09-18

**Authors:** Alicia C. Strtak, Jacob L. Perry, Mark N. Sharp, Alexandra L. Chang-Graham, Tibor Farkas, Joseph M. Hyser

**Affiliations:** a Alkek Center for Metagenomic and Microbiome Research, Department of Molecular Virology and Microbiology, Baylor College of Medicine, Houston, Texas, USA; b Department of Pathobiological Sciences, Louisiana State University School of Veterinary Medicine, Baton Rouge, Louisiana, USA; c Louisiana Animal Disease Diagnostic Laboratory, Baton Rouge, Louisiana, USA; d Texas Medical Center Summer Research Internship Program, Augustana College, Rock Island, Illinois, USA; Wake Forest University

**Keywords:** GCaMP, calcium, calicivirus, viroporin

## Abstract

Enteric viruses in the *Caliciviridae* family cause acute gastroenteritis in humans and animals, but the cellular processes needed for virus replication and disease remain unknown. A common strategy among enteric viruses, including rotaviruses and enteroviruses, is to encode a viral ion channel (i.e., viroporin) that is targeted to the endoplasmic reticulum (ER) and disrupts host calcium (Ca^2+^) homeostasis. Previous reports have demonstrated genetic and functional similarities between the nonstructural proteins of caliciviruses and enteroviruses, including the calicivirus NS1-2 protein and the 2B viroporin of enteroviruses. However, it is unknown whether caliciviruses alter Ca^2+^ homeostasis for virus replication or whether the NS1-2 protein has viroporin activity like its enterovirus counterpart. To address these questions, we used Tulane virus (TV), a rhesus enteric calicivirus, to examine Ca^2+^ signaling during infection and determine whether NS1-2 has viroporin activity that disrupts Ca^2+^ homeostasis. We found that TV increases Ca^2+^ signaling during infection and that increased cytoplasmic Ca^2+^ levels are important for efficient replication. Further, TV NS1-2 localizes to the endoplasmic reticulum, the predominant intracellular Ca^2+^ store, and the NS2 region has characteristics of a viroporin domain (VPD). NS1-2 had viroporin activity in a classic bacterial functional assay and caused aberrant Ca^2+^ signaling when expressed in mammalian cells, but truncation of the VPD abrogated these activities. Together, our data provide new mechanistic insights into the function of the NS2 region of NS1-2 and support the premise that enteric viruses, including those within *Caliciviridae*, exploit host Ca^2+^ signaling to facilitate their replication.

**IMPORTANCE** Tulane virus is one of many enteric caliciviruses that cause acute gastroenteritis and diarrheal disease. Globally, enteric caliciviruses affect both humans and animals and amass >65 billion dollars per year in treatment and health care-associated costs, thus imposing an enormous economic burden. Recent progress has resulted in several cultivation systems (B cells, enteroids, and zebrafish larvae) to study human noroviruses, but mechanistic insights into the viral factors and host pathways important for enteric calicivirus replication and infection are still largely lacking. Here, we used Tulane virus, a calicivirus that is biologically similar to human noroviruses and can be cultivated by conventional cell culture, to identify and functionally validate NS1-2 as an enteric calicivirus viroporin. Viroporin-mediated calcium signaling may be a broadly utilized pathway for enteric virus replication, and its existence within caliciviruses provides a novel approach to developing antivirals and comprehensive therapeutics for enteric calicivirus diarrheal disease outbreaks.

## Abstract (precis)

Tulane virus is one of many enteric caliciviruses that cause acute gastroenteritis and diarrheal disease. Globally, enteric caliciviruses affect both humans and animals and amass >65 billion dollars per year in treatment and health care-associated costs, thus imposing an enormous economic burden. Recent progress has resulted in several cultivation systems (B cells, enteroids, and zebrafish larvae) to study human noroviruses, but mechanistic insights into the viral factors and host pathways important for enteric calicivirus replication and infection are still largely lacking. Here, we used Tulane virus, a calicivirus that is biologically similar to human noroviruses and can be cultivated by conventional cell culture, to identify and functionally validate NS1-2 as an enteric calicivirus viroporin. Viroporin-mediated calcium signaling may be a broadly utilized pathway for enteric virus replication, and its existence within caliciviruses provides a novel approach to developing antivirals and comprehensive therapeutics for enteric calicivirus diarrheal disease outbreaks.

## INTRODUCTION

The *Caliciviridae* family consists of small, nonenveloped single-stranded RNA viruses with 11 recognized genera (1, 2). Caliciviruses (CVs) infect a wide array of hosts and have importance in medical, veterinary, and agricultural fields (3). Of particular importance are human noroviruses (HuNoVs), which are the leading cause of acute gastroenteritis (AGE) in every age group, and can cause life-threatening illness in the young, immunocompromised, and elderly subpopulations (4–7). Estimates show that every individual will experience at least five symptomatic norovirus infections in their life (8), which underlines the need for antiviral drugs, vaccines, or antidiarrheal therapies for HuNoV infection (9, 10). However, many aspects of calicivirus pathogenesis, including that of HuNoV, remain uncharacterized, which represents a challenge to developing effective therapies (4, 6, 9). One strategy to address this challenge is to study other enteric caliciviruses, such as porcine sapoviruses and rhesus enteric caliciviruses (*Recovirus*). Recoviruses are members of a newly approved genus of CVs initially identified in stool samples from rhesus macaques, of which Tulane virus (TV) is the prototype strain (11, 12). While recoviruses constitute a separate genus, these viruses are most closely related to HuNoVs and studies of TV show that it retains both biologic and genetic similarities to HuNoVs, including genomic organization, tissue tropism (intestinal epithelia), and clinical presentation (self-limiting vomiting and diarrhea) (1, 11–13). Furthermore, TV robustly replicates in cell culture in monkey kidney cell lines (e.g., LLC-MK2 cells), which facilitates investigation into the host pathways exploited by TV during infection. This makes TV an excellent model system to identify host signaling pathways broadly exploited by caliciviruses for replication and pathogenesis.

Like some other caliciviruses, TV has three main open reading frames (ORFs), with ORF1 encoding the nonstructural proteins (NS1-7), and ORFs 2 and 3 encoding the capsid proteins VP1 (ORF2) and VP2 (ORF3) (10, 14, 15). During replication, ORF1 is synthesized into the polyprotein, which is subsequently cleaved by the viral protease NS6 to produce six nonstructural proteins that orchestrate viral replication (4, 12, 14–16). Outside of murine norovirus (MNV), the roles of the NS1-2 protein (N-terminal protein) during viral replication and pathogenesis are not well characterized. However, work with MNV-1 NS1-2 may elucidate some of the functions NS1-2 performs in other calicivirus infections. For example, full-length MNV-1 NS1-2 is cleaved by caspase-3 during infection, which has been shown to mediate intestinal epithelial tropism, spread, and persistence (17). Additionally, MNV NS1, the N-terminal portion of NS1-2, antagonizes the interferon pathway (17–19). Recombinant expression of NS1-2 from feline calicivirus (FCV), MNV, and HuNoV GII.4 shows that the protein traffics to the endoplasmic reticulum (ER), concentrates perinuclearly, colocalizes with the ER-resident protein calnexin, and contains C-terminal hydrophobic sequences (20–23). In contrast, Norwalk virus (GI.1) NS1-2 (p48) was primarily found in the Golgi apparatus and implicated in disrupting ER-to-Golgi trafficking (24, 25). The similarities in ER/Golgi membrane association and domain organization of NS1-2 from different viruses suggest that NS1-2 may have a conserved function among caliciviruses.

The ER, and to a lesser extent, the Golgi apparatus are important intracellular calcium (Ca^2+^) storage organelles, with the ER Ca^2+^ concentration as high as 1 mM (26, 27). As a ubiquitous secondary messenger, Ca^2+^ is at the epicenter of many cellular processes, and host machinery tightly regulates Ca^2+^ levels to ensure low (nanomolar) cytoplasmic Ca^2+^ concentrations at cellular rest (27–33). Importantly, Ca^2+^ signaling regulates several aspects of viral life cycles, including entry, genome replication, and release (31, 34–36). To exploit Ca^2+^ signaling, many viruses express an ion channel (i.e., viroporin) to dysregulate Ca^2+^ homeostasis in order to usurp Ca^2+^-dependent host processes (31, 37–40). The best-characterized Ca^2+^-disrupting viroporins are the nonstructural protein 4 (NSP4) from rotavirus (RV) and the 2B nonstructural protein of enteroviruses (EVs) and some other picornaviruses (37, 38, 41–45). Like all bona fide viroporins, NSP4 and 2B have canonical biophysical motifs, including being oligomeric, having an amphipathic α-helix that forms the pore, and a cluster of basic residues that facilitate membrane insertion (38–40, 43–45). While no study has specifically looked at whether caliciviruses dysregulate Ca^2+^ signaling or have a viroporin, they belong to the picornavirus-like superfamily of positive-sense RNA viruses, among which there is considerable positional homology of the cognate proteins of the nonstructural polyprotein (24, 46, 47). Within this rubric, the picornavirus 2AB region constitutes the positional homolog of the calicivirus NS1-2 protein, and several sequence motifs in NS1 are conserved in the 2A protein of some picornaviruses (24). While no functional homology between EV 2B and the NS2 region of NS1-2 has yet been identified, it is tempting to speculate that NS1-2 may have viroporin activity and dysregulate host Ca^2+^ signaling analogous to that of EV 2B.

In this study, we investigated the role of Ca^2+^ signaling in TV replication and whether TV NS1-2 has viroporin activity that can dysregulate Ca^2+^ homeostasis. Using long-term live-cell Ca^2+^ imaging, we sought to determine whether TV infection causes aberrant Ca^2+^ signaling during infection and identify the cellular Ca^2+^ pools critical for the TV-induced Ca^2+^ signaling. Finally, we tested TV NS1-2 for viroporin activity and determined whether the putative NS1-2 viroporin domain caused aberrant Ca^2+^ signaling similar to TV infection.

## RESULTS

### TV infection disrupts host calcium signaling kinetics in LLC-MK2 cells.

**FIG 1:**
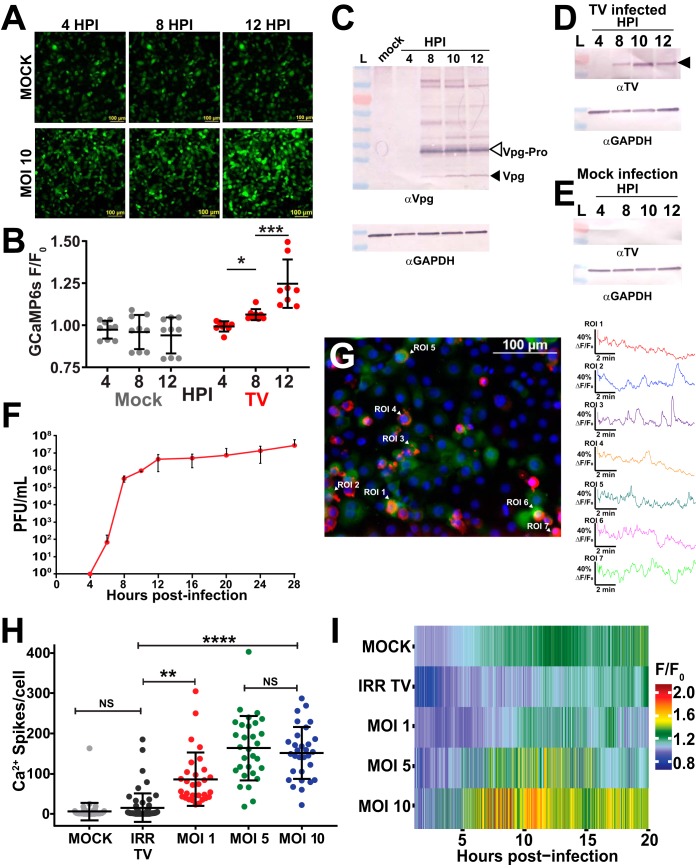
TV infection disrupts host calcium signaling kinetics in LLC-MK2 cells. (A) Representative images at early (4 h postinfection [HPI]), onset (8 HPI), and late (12 HPI) stages of mock-infected (top) and TV-infected (bottom) LLC-MK2 GCaMP6s cells. (B) Quantification of GCaMP6s fluorescence from panel A. (C and D) Western blots of TV-infected lysates for nonstructural protein Vpg (C) and structural protein VP1 (D) confirm that aberrant Ca^2+^ signaling in infected cells coincides with both structural and nonstructural protein synthesis. Mature Vpg in panel C is indicated by a black arrowhead, and the major band (open arrowhead) represents the Vpg-Pro precursor (∼30 kDa). L, lysate; αVpg, anti-Vpg. (E) Western blot of mock lysates for structural protein VP1. (F) One-step growth curve for TV at a low MOI (MOI of 1) shows that virus replication is concomitant with viral protein synthesis (C and D) and with changes in Ca^2+^ signaling (A). (G) Image from overlay of anti-Vpg staining (red) onto short (10-min) continuous imaging runs of TV-infected cells (MOI of 5) at 12 HPI. Accompanying Ca^2+^ cell traces (right) show the dynamic increases in cytosolic Ca^2+^ in infected cells. ROI, region of interest. (H) Compared to mock-infected cells, TV-infected cells have an increased number of Ca^2+^ spikes per cell that increases in an infectious dose-dependent manner, saturating at an MOI of 5. IRR TV, gamma-irradiated TV. (I) Heatmap data suggest that Ca^2+^ signaling increases with infectious dose and that a higher MOI disrupts host Ca^2+^ signaling earlier in infection and sustains this aberrant Ca^2+^ signaling throughout. Mock-infected and irradiated TV have similar heatmap profiles, suggesting that replication-competent virus is required to drive these changes in Ca^2+^ signaling. Data are shown as means ± standard deviations (SD) (error bars). Values that are significantly different are indicated by a bar and asterisks as follows: *, *P* < 0.05; **, *P* < 0.01; ***, *P* < 0.001; ****, *P* < 0.0001. Values that are not significantly different (NS) are also indicated. *N* ≥ 3 for each experiment, except the one-step growth curve, which was *N* = 2 with three replicates per experiment.

Ca^2+^ is a ubiquitous secondary messenger and many enteric viruses (e.g., RVs and EVs) require elevated cytosolic Ca^2+^ to facilitate replication (31, 37–40, 43, 44). To determine whether TV causes aberrant Ca^2+^ signaling like other enteric viruses, we examined whether Ca^2+^ signaling dynamics changed during TV infection. We infected LLC-MK2 cells stably expressing GCaMP6s (MK2-G6s) with different infectious doses (multiplicities of infection [MOI] of 1, 5, and 10) or γ-irradiated inactivated TV and performed live-cell fluorescence microscopy during the infection. GCaMP6s is a green fluorescent protein (GFP)-based genetically encoded Ca^2+^ indicator that reports changes in cytosolic Ca^2+^ as an increase in fluorescence (48). TV-infected MK2-G6s cells show increased cytoplasmic Ca^2+^ levels (Fig. 1A) beginning at roughly 8 h postinfection (HPI) (MOI of 10), and quantitation of the GCaMP6s signal shows a significant increase at 8 and 12 HPI (Fig. 1B). This is illustrated in the time-lapse movie of the infection (see Movie S1 in the supplemental material). The observed increase in Ca^2+^ signaling coincides with the synthesis of TV nonstructural proteins, assessed by Western blotting using anti-Vpg (Fig. 1C, black arrowhead) and anti-TV (Fig. 1D, black arrowhead) antisera, which show increased TV protein production between 8 and 12 HPI, but no detection of Vpg or VP1 in mock lysates (Fig. 1C and E). Further, based on a one-step growth curve, the increased cytosolic Ca^2+^ also coincides with the onset of progeny virus production, which occurs between 6 and 8 HPI (Fig. 1F). The increases in cytosolic Ca^2+^ were dynamic during TV infection (Movie S2). We noted that in infected cells, changes in cytosolic Ca^2+^ occurred through an increased number of discrete Ca^2+^ signals, much like what we recently observed in RV-infected cells (Fig. 1G) (66). We refer to these high-amplitude, transient Ca^2+^ signals as “Ca^2+^ spikes” and quantitated the number of Ca^2+^ spikes per cell during infection. Compared to uninfected controls, TV-infected cells have significantly more Ca^2+^ spikes/cell, but cells inoculated with γ-irradiated TV did not exhibit increased Ca^2+^ signaling (Fig. 1H). Together, these data indicate that increased Ca^2+^ signaling requires replication-competent virus and occurs later during infection, well after entry has occurred. Additionally, Ca^2+^ signaling in infected cells increases in an infectious-dose-dependent manner, saturating at an MOI of 5 (Fig. 1H). To visualize the aberrant Ca^2+^ signaling induced by TV, we generated heatmaps plotting normalized GCaMP6s fluorescence over time (Fig. 1I). Heatmap data show an increased number and magnitude of Ca^2+^ signals and that cytosolic Ca^2+^ levels change earlier and more frequently throughout infection as the infectious dose increases (Fig. 1I). The heatmaps also show that MK2-G6s cells inoculated with γ-irradiated TV do not have increased Ca^2+^ signaling compared to mock-inoculated cells (Fig. 1I), consistent with the lack of increased Ca^2+^ spikes (Fig. 1H). Taken together, these data suggest that, like other enteric viruses, TV disrupts host Ca^2+^ signaling kinetics during infection.

10.1128/mSphere.00506-19.5 MOVIE S1 Representative imaging run of LLC-MK2 GCaMP6s cells infected at a wide infectious dose (MOIs of 1, 5, and 10) compared to irradiated TV. The movies qualitatively show that irradiated virus does not induce aberrant Ca^2+^ signaling but that replication-competent virus does. Quantification of this imaging run can be seen in Fig. 1. Download Movie S1, MPG file, 7.8 MB. Copyright © 2019 Strtak et al. 2019 Strtak et al. This content is distributed under the terms of the Creative Commons Attribution 4.0 International license.

10.1128/mSphere.00506-19.6 MOVIE S2 IF staining for TV nonstructural protein anti-Vpg overlaid onto short, continuous imaging run of TV-infected cells (MOI of 5) at 12 HPI. TV-infected cells (red) exhibit dynamic changes in cytosolic Ca^2+^. Changes manifest as discrete Ca^2+^ spikes which can be observed as large changes in cytosolic fluorescence. Download Movie S2, MPG file, 9.6 MB. Copyright © 2019 Strtak et al. 2019 Strtak et al. This content is distributed under the terms of the Creative Commons Attribution 4.0 International license.

### Intracellular Ca^2+^ is critical for TV replication.

**FIG 2:**
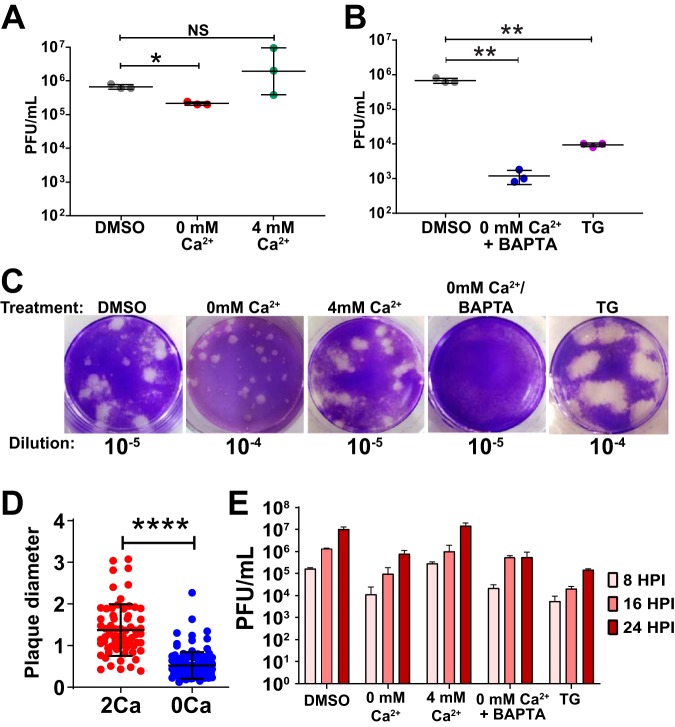
Intracellular calcium is critical for TV replication. (A) Buffering out extracellular calcium hinders TV replication, significantly reducing the total plaque-forming units (PFU). In contrast, excess extracellular Ca^2+^ (4 mM Ca^2+^, right) does not impact replication. (B) Buffering intracellular calcium reduces replication. Depleting ER calcium stores with the SERCA inhibitor thapsigargin (TG), and reducing cytoplasmic Ca^2+^ with BAPTA-AM significantly reduce TV infectious yield (PFU/ml). (C) Representative images of plaques under normal Ca^2+^ conditions (2 mM) and reduced Ca^2+^ (0 mM Ca^2+^/BAPTA-AM, TG), conditions. The treatment condition is listed above each image, while the dilution each image represents is listed below each image. (D) Diameter of plaques from TV infections cultured in 2 mM Ca^2+^ (2Ca) or 0 mM Ca^2+^ (0Ca). (E) Partial one-step growth curve data altering free intracellular (IC) and extracellular (EC) Ca^2+^. TV replication is stunted in Ca^2+^-free IC and EC conditions (0 mM Ca^2+^, 0 mM Ca^2+^/BAPTA-AM). Inhibiting ER Ca^2+^ replenishment with thapsigargin (TG) also blunts replication, suggesting that IC Ca^2+^ stores are critical for TV replication. Data shown are means ± SD. *, *P* < 0.05; **, *P* < 0.001; ****, *P* < 0.0001; NS, not significantly different. *N* ≥ 3 for each experiment.

Since we observed aberrant Ca^2+^ signaling during TV infection, we sought to determine whether Ca^2+^ was involved in TV replication. To test this, we manipulated extracellular and intracellular Ca^2+^ levels and determined the effects on TV yield. Doubling the extracellular Ca^2+^ concentration (∼4 mM) did not affect TV yield (Fig. 2A, right). In contrast, TV propagated in Ca^2+^-free media significantly reduced total yield (Fig. 2A, middle). Interestingly, plaques of TV propagated in Ca^2+^-free media were significantly smaller than that propagated in normal media, even though the plaque assay titrations were performed in normal media (Fig. 2C and D). Next, to investigate the role of intracellular Ca^2+^ during infection, we treated LLC-MK2 cells with BAPTA-AM, which chelates cytosolic Ca^2+^ and therefore buffers cytosolic Ca^2+^ (49, 50). TV replication in Ca^2+^-free media supplemented with BAPTA-AM (0 mM Ca^2+^ + BAPTA) was reduced up to 4 log units (Fig. 2B), which was a greater inhibition than Ca^2+^-free media alone (Fig. 2A versus Fig. 2B). We next sought to determine whether intracellular Ca^2+^ stores are important for TV replication by testing the effect of thapsigargin (TG) on TV replication. TG is an inhibitor of sarco/endoplasmic reticulum (SERCA) Ca^2+^ ATPase, which pumps cytosolic Ca^2+^ into the ER to help maintain ER Ca^2+^ stores. We treated TV-infected cells with TG and measured TV yield as described in Materials and Methods and found that TV replication is ∼3 log units lower in TG-treated cells than in dimethyl sulfoxide (DMSO)-treated cells (Fig. 2B). Finally, we tested these different manipulations of extracellular or intracellular Ca^2+^ on TV yield at different time points during infection (8, 16, and 24 HPI) (Fig. 2E). These studies confirmed that reduction of extracellular Ca^2+^ or treatment with TG significantly inhibited total virus replication; however, the rate of progeny virus production was not substantially reduced. Together, the replication assays demonstrate that intracellular Ca^2+^ levels facilitate TV replication and that the ER Ca^2+^ store is particularly important for robust virus production.

### TV-induced Ca^2+^ signaling requires ER Ca^2+^ stores.

**FIG 3:**
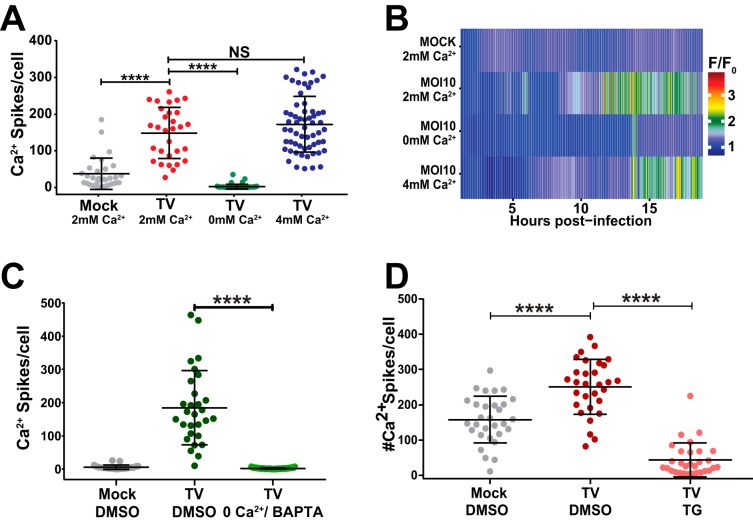
TV-induced Ca^2+^ signaling requires ER Ca^2+^ stores. (A) Ca^2+^-free media reduces Ca^2+^ signaling in TV-infected cells, suggesting that Ca^2+^ signaling is activated during infection. (B) TV infection in 0 mM Ca^2+^ phenocopies mock Ca^2+^ traces in heatmap data, suggesting that extracellular (EC) Ca^2+^ facilitates TV infection. (C) Intracellular Ca^2+^ chelator BAPTA-AM abrogated TV-induced Ca^2+^ signaling. BAPTA-AM-treated TV-infected cells (light green) returns Ca^2+^ signaling to uninfected levels (gray). (D) Depleting ER Ca^2+^ with SERCA blocker thapsigargin (TG) significantly reduces TV-induced Ca^2+^ signaling (pink), suggesting that ER Ca^2+^ stores are a key source of Ca^2+^ leveraged during infection. Data shown are means ± SD. ****, *P* < 0.0001; NS, not significant. *N* ≥ 3 for each experiment.

We next sought to determine the effects that the manipulations to extracellular and intracellular Ca^2+^ had on the TV-induced Ca^2+^ signaling exhibited in Fig. 1. We altered extracellular and intracellular Ca^2+^ concentrations as described in Materials and Methods and performed live Ca^2+^ imaging of mock-infected and TV-infected MK2-G6s cells. TV-infected cells in 2 mM Ca^2+^ (normal media) exhibited increased Ca^2+^ signaling, as observed above (Fig. 3A). Supplementing media with additional extracellular Ca^2+^ (4 mM Ca^2+^ total) did not further increase the Ca^2+^ spikes, but removing extracellular Ca^2+^ abolished the TV-induced Ca^2+^ spikes (Fig. 3A). Using heatmaps, we plotted the relative change in GCaMP6s fluorescence over time and observed increased signaling starting at ∼8 HPI in both the 2 mM Ca^2+^ and 4 mM Ca^2+^ conditions (Fig. 3B). Further, the heatmaps show that infected cells in Ca^2+^-free media have a signaling profile that phenotypically mimics uninfected controls (Fig. 3B). Like the results obtained in replication assays, buffering cytoplasmic Ca^2+^ using BAPTA-AM reduced the number of Ca^2+^ spikes per cell to a level comparable to that of mock-infected cells (Fig. 3C and Movie S3). Similarly, blocking the ER SERCA pump with TG significantly reduces TV-induced Ca^2+^ signaling (Fig. 3D), supporting replication data and demonstrating that ER Ca^2+^ stores are a critical source of Ca^2+^ for enhancing replication.

10.1128/mSphere.00506-19.7 MOVIE S3 Representative imaging run of LLC-MK2 GCaMP6s cells infected with TV (MOI of 10) (middle) compared to TV-infected cells treated with 0Ca^2+^/BAPTA (right). BAPTA treatment during TV infection abrogates TV-induced Ca^2+^ signaling and results in a Ca^2+^ signaling profile that mirrors mock LLC-MK2 cells (left). Quantification of this imaging run can be seen in Fig. 3. Download Movie S3, MPG file, 12.0 MB. Copyright © 2019 Strtak et al. 2019 Strtak et al. This content is distributed under the terms of the Creative Commons Attribution 4.0 International license.

### Tulane virus NS1-2 is targeted to the ER membrane.

**FIG 4:**
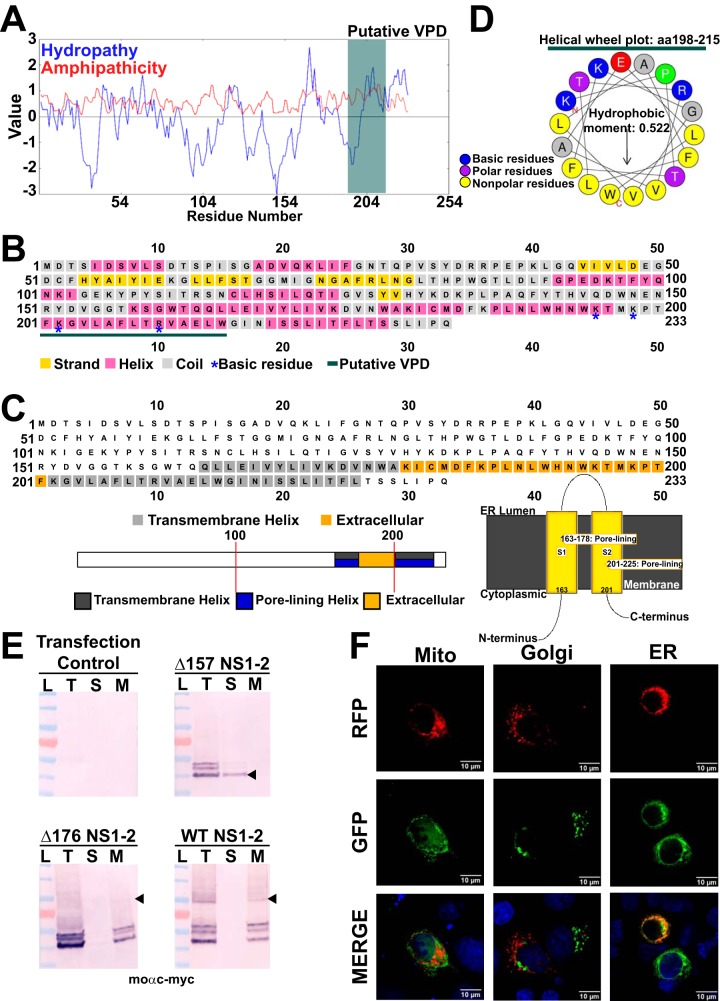
Tulane virus NS1-2 is targeted to the ER membrane. (A) Predictive modeling of TV NS1-2 reveals that it has essential features of bona fide viroporins. Kyte-Doolittle hydropathy plots predict an amphipathic moment from amino acids 195 to 212 (aa195-212) (dark green bar), consistent with alpha-helical structure required for channel formation. (B) PSIPred secondary structure algorithms predict that the C terminus of NS1-2 is helical in nature, with the putative viroporin domain (VPD) (dark green bar) contained to helices. (C, top) PSIPred membrane topology predictions suggest that NS1-2 has two transmembrane helices (gray squares). PSIPred algorithms predicting transmembrane helices suggest that NS1-2 transmembrane domains are pore lining (bottom left) and propose a model of membrane insertion and orientation where the putative VPD (aa195-212) comprises the pore-lining helix (bottom right). (D) Helical wheel plot generated from the NS1-2 amphipathic segment (dark green bar) shows clustered basic residues (blue circles) and a hydrophobic moment of 0.522 from aa198-215, coinciding with the putative VPD. (E) Mammalian expressed full-length RFP-NS1-2 and RFP NS1-2 Δ176 are membrane associated, but RFP NS1-2 Δ157 is not. Both the total fraction (T) and membrane pellets (M) extracted with 1% SDS contain RFP-NS1-2 and Δ176, but centrifuged supernatant (S) does not, suggesting that RFP-NS1-2 and Δ176 are membrane-associated proteins. In contrast, the supernatant contains RFP-NS1-2 Δ157. Further, immunoblot assays run under nonreducing conditions show that full-length RFP-NS1-2 and Δ176 oligomerize (black arrowheads). No detection of NS1-2 observed in transfection control lysates. L, lysate; moαmyc, anti-myc monoclonal antibody. (F) Cotransfection experiments using intracellular markers for predominant intracellular Ca^2+^ stores mitochondria (Mito), Golgi apparatus, and endoplasmic reticulum (ER) to determine whether TV NS1-2 associated with any intracellular organelle(s). Based on deconvolution microscopy data, RFP-NS1-2 localized to the ER (right), but not with the Golgi apparatus (middle). RFP-NS1-2 did not localize to the mitochondria (left) (*N* ≥ 2). *N* ≥ 3 for immunoblot experiments.

Our data indicate that TV activates aberrant Ca^2+^ signaling involving the ER Ca^2+^ store, much like the dysregulation of Ca^2+^ homeostasis by other enteric viruses observed in RV and EV infections. Both RV and EV encode a viroporin, or viral ion channel, that targets the ER Ca^2+^ store to activate aberrant Ca^2+^ signaling pathways that are critical for virus replication (36, 37, 39, 40, 43, 44). Viroporins are integral membrane proteins that have some common characteristics, including being oligomeric, having an amphipathic α-helix that serves as the channel lumen through the membrane, and a cluster of basic amino acid residues that facilitate insertion into the membrane (25, 35, 36, 40, 51). Previous work with NS1-2 from several different caliciviruses shows that it is membrane associated and localizes primarily to the ER (18–21, 25) and/or Golgi apparatus (20, 21, 23, 24). Thus, we hypothesized that calicivirus NS1-2 could be a viroporin involved in the aberrant Ca^2+^ signaling we observed during TV infection. Notably, the calicivirus NS2 domain is the positional homolog of the EV 2B viroporin (see Fig. S1A in the supplemental material). This is potentially significant because previous studies have found conserved functional characteristics between the positional homologs of the other nonstructural proteins (21, 23, 24, 47, 51–54), and functional homology between EV 2AB and human norovirus (HuNoV) GII.4 NS1-2 (21, 24). Additionally, when performing multiple-sequence alignments of other calicivirus NS1-2s, we found that the C-terminal domain (CTD) is highly conserved, particularly in the putative viroporin domain (Fig. S1B). To determine whether TV NS1-2 has viroporin-like characteristics, we examined TV NS1-2 for viroporin motifs. First, we performed a Kyte-Doolittle plot to detect hydrophobic regions of NS1-2 and an amphipathicity plot to identify potential amphipathic domains (Fig. 4A). We found that amino acids 195 to 215 (aa195-215) (Fig. 4A, dark green box) in the CTD of NS1-2 has a high amphipathic moment. We then used PSIPred (55) to model NS1-2 predicted secondary structure (Fig. 4B). Output from this analysis suggested that the NS1-2 CTD was predominantly comprised of α-helices (Fig. 4B, pink residues), and accompanying confidence scores for prediction of these C-terminal helices were ≥75% (Fig. S2). Interestingly, the region of peak amphipathicity (Fig. 4A) was located within one of the PSIPred helix predictions of the CTD (Fig. 4B, dark green bar) and contained clustered basic residues (blue asterisks), two key features of viroporins. Additionally, NS1-2 topology modeling identified two putative transmembrane domains (TMDs): the first (TMD1) from aa164-179, and the second (TMD2) from aa202-225 (Fig. 4C, top). The membrane topology schematic indicated that both TMD1 and TMD2 had predicted pore-lining regions within their helices (Fig. 4C, bottom left). To explore this, we used HeliQuest (56) to generate a helical wheel diagram for TMD2 (aa198-215), since TMD2 had the clustered basic residues common among viroporins. The helical wheel shows that TMD2 is highly amphipathic with clear polar and nonpolar faces to the helix (Fig. 4D). The calculated hydrophobic moment for TMD2 is 0.522, supporting the above amphipathicity predictions (Fig. 4A). Given the results of these computational studies, we predicted that NS1-2 TMD2 (aa195-215) is a viroporin domain and set out to test this prediction experimentally.

10.1128/mSphere.00506-19.1 FIG S1 (A) Aligning the nonstructural regions of both the TV and EV genome reveals that TV NS1-2 is the positional homolog of enterovirus (EV) 2B, a *bona fide* viroporin. (B) Multiple-sequence alignment results of NS1-2 sequences from various calicivirus strains. Sequence alignment results through T-Coffee show that the C-terminal domain (CTD) of NS1-2 is well conserved, particularly in the putative viroporin domain. Download FIG S1, PDF file, 2.84 MB. Copyright © 2019 Strtak et al. 2019 Strtak et al. This content is distributed under the terms of the Creative Commons Attribution 4.0 International license.

10.1128/mSphere.00506-19.2 FIG S2 Confidence of prediction values of secondary structure for NS1-2 amino acid residues. Computational prediction for the viroporin domain (VPD) of NS1-2 places it between aa195-215, which is predominantly helical, with ≥75% confidence of prediction throughout most of the C-terminal domain (aa160-232) (dark blue bars = conf). Download FIG S2, PDF file, 0.3 MB. Copyright © 2019 Strtak et al. 2019 Strtak et al. This content is distributed under the terms of the Creative Commons Attribution 4.0 International license.

First, we tested whether TV NS1-2 was an integral membrane protein and whether it localized to the ER similar to NS1-2 from other caliciviruses. To do so, we generated bacterial and mammalian expression vectors of full-length NS1-2. For mammalian expression vectors, we N-terminally fused full-length NS1-2 to mRuby3 (henceforth referred to as RFP-NS1-2 [RFP stands for red fluorescent protein]). From these constructs, we generated two truncation mutants of wild-type NS1-2 in both mammalian and bacterial expression vectors: the first, NS1-2 Δ176, was predicted to have TMD1 but lack the viroporin domain, and the second, NS1-2 Δ157, was predicted to lack both TMD1 and the VPD. We then transfected wild-type, full-length (WT) RFP-NS1-2, RFP-NS1-2 Δ157, and RFP-NS1-2 Δ176 into HEK 293FT cells and harvested cell suspensions next day. Samples after cell lysis, sonication, and fractionation were collected for SDS-PAGE Western blots. We found both Δ176 and WT TV NS1-2 in the total fraction (T) and membrane pellets (M), but not in the supernatant (S), suggesting that TMD1 mediates membrane association (Fig. 4E). Additionally, in the nonreducing, unboiled conditions used, oligomers of both Δ176 and WT RFP-NS1-2 were detected by Western blotting (Fig. 4E, black arrowheads). Similar results were obtained from membrane fractionation of analogous bacterially expressed NS1-2 constructs (Fig. S3). Using the mammalian expression vectors of RFP-NS1-2, we performed colocalization assays with fluorescent markers of the ER, Golgi apparatus, and mitochondria. RFP-NS1-2 showed no colocalization with the mitochondria or Golgi apparatus (Fig. 4F). In contrast, RFP-NS1-2 strongly colocalized with the ER-GFP marker (Fig. 4F), indicating that, like NS1-2 from other caliciviruses and EV 2B and RV NSP4, TV NS1-2 traffics to the ER membrane.

10.1128/mSphere.00506-19.3 FIG S3 Immunoblot analysis of cell fractionation studies of bacterially expressed full-length WT NS1-2 and the Δ176 and Δ157 truncation mutants. Samples of total cell lysate (T), the soluble fraction (S), or the membrane fraction (M) were resolved by SDS-PAGE and detected by immunoblot using an anti-6xHis antibody. Oligomers of NS1-2 and the truncations are indicated by arrowheads. Download FIG S3, PDF file, 1.46 MB. Copyright © 2019 Strtak et al. 2019 Strtak et al. This content is distributed under the terms of the Creative Commons Attribution 4.0 International license.

10.1128/mSphere.00506-19.4 FIG S4 Validation of antisera against TV Vpg (A) and purified TV particles (B). (A) Bacterial expression vectors for the indicated TV nonstructural proteins were generated using the pET46 vector, which includes a N-terminal 6xHis tag. E. coli BL21(DE3)pLysS cells transformed with the indicated expression vector were induced for 3 h with 1 mM IPTG or left uninduced (UI), cells were collected and lysed in PBS supplemented with 1% SDS. Lysates were resolved by SDS-PAGE, and proteins were detected by the anti-Vpg antisera (left) or anti-6xHis antibody (right). The positions of His-Vpg monomer and oligomer are indicated by arrowheads. (B) Validation of the specificity of the anti-TV antisera using mock-inoculated or TV-infected LLC-MK2 cell lysates. Blots were detected by the anti-TV antisera (left) or anti-GAPDH antibody (right). (C) Table of primers used to construct the bacterial and mammalian expression vectors. Download FIG S4, PDF file, 1.82 MB. Copyright © 2019 Strtak et al. 2019 Strtak et al. This content is distributed under the terms of the Creative Commons Attribution 4.0 International license.

### TV NS1-2 has viroporin activity that disrupts Ca^2+^ signaling.

**FIG 5:**
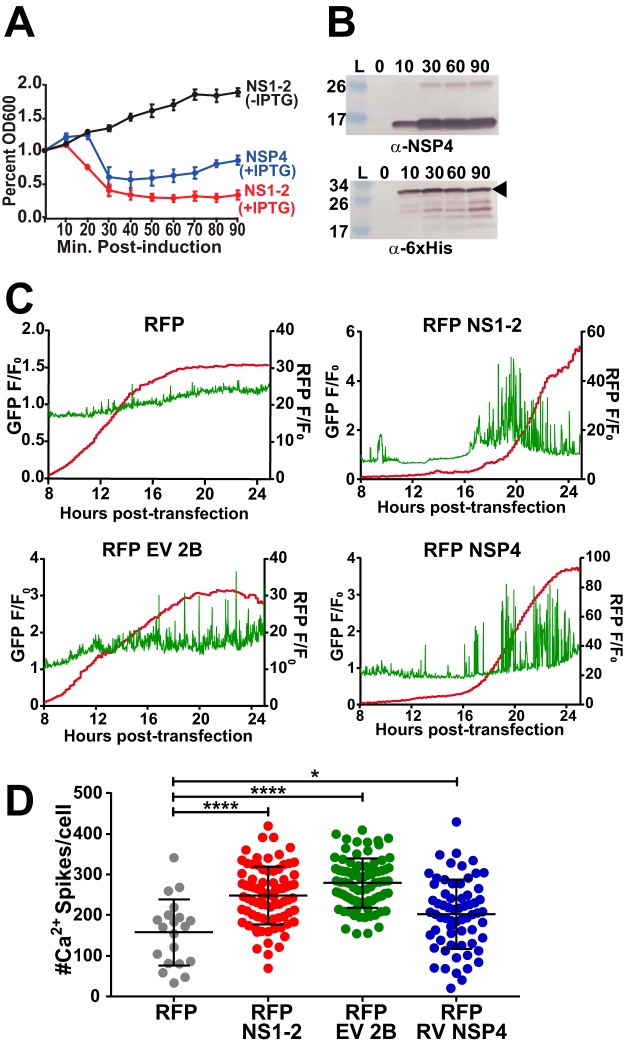
TV NS1-2 has viroporin activity that disrupts Ca^2+^ signaling in mammalian cells. (A) Inducing TV NS1-2 in the lysis assay strongly reduces optical density similar to rotavirus NSP4, the positive control for viroporin activity. (B) Western blot data to verify protein expression during the lysis assay for TV NS1-2 (bottom, black arrowhead) and RV NSP4 (top). (C and D) Mammalian recombinant RFP-NS1-2 increases the number (D) and amplitude (C, top row, right) of Ca^2+^ spikes when transfected into cells similar to RV NSP4 and EV 2B, the viroporin controls for these experiments. Data shown are means ± SD from ≥8 fields of view. *, *P* < 0.05; ****, *P* < 0.0001. *N* ≥ 3 for each experiment.

Since our predictive modeling suggested that NS1-2 met the biophysical requirements for a viroporin and our live-cell Ca^2+^ imaging data exhibited large changes in cytosolic Ca^2+^ during TV infection, we tested whether NS1-2 has viroporin activity. We performed the Escherichia coli lysis assay, which is a classical viroporin functional assay, wherein viroporin expression by E. coli BL21(DE3)pLysS results in permeabilization of the inner membrane, resulting in T7 lysozyme-mediated cell lysis (42). This assay has been used to identify and initially characterize many viroporins (37, 57, 58). We expressed full-length HisNS1-2 in BL21(DE3)pLysS cells and measured optical density (OD) over time after protein induction with IPTG. For the lysis assay, strong viroporin activity is characterized by large decreases in OD over time, whereas no viroporin activity is characterized by increases in OD over time. Our results show that induced NS1-2 has strong viroporin activity, similar to that of RV NSP4, our positive control for viroporin activity (Fig. 5A). We see no changes in OD over time for uninduced NS1-2, indicating that histidine-tagged NS1-2 (HisNS1-2) viroporin activity correlated with protein expression, detected by immunoblotting for the 6×His tag (Fig. 5B). We then asked whether recombinant expression of RFP-NS1-2 alone increases Ca^2+^ signaling in MK2-G6s cells. To test this, we transfected MK2-G6s cells with mammalian expression vectors for RFP-NS1-2 as well as RFP-NSP4 and RFP-EV 2B, our positive controls for viroporin-mediated Ca^2+^ signaling. Expressing RFP-tagged viroporins in MK2-G6s cells significantly increases both the number and amplitude of Ca^2+^ spikes. However, this was not observed in cells expressing RFP alone, as illustrated by the representative single-cell traces (Fig. 5C and Movie S4). As described above, we quantitated the number of Ca^2+^ spikes and confirmed that recombinant expression of RFP-NS1-2 increased the number of Ca^2+^ spikes per cell approximately twofold, similar to that of EV 2B and RV NSP4 (Fig. 5D). Taken together, our results demonstrate that TV NS1-2 has viroporin activity in the lysis assay, similar to bona fide viroporins, and causes aberrant host Ca^2+^ signaling when expressed in mammalian cells.

10.1128/mSphere.00506-19.8 MOVIE S4 Representative signaling from viroporin transfection experiments in LLC-MK2 GCaMP6s cells. The movies show that cells exhibit aberrant Ca^2+^ signaling upon expression of RFP-tagged viroporins. TV NS1-2 (left) induces aberrant Ca^2+^ signaling in cells with similar activity to RV NSP4 (middle) and EV 2B (right). Quantification of this imaging run can be seen in Fig. 5. Download Movie S4, MPG file, 14.1 MB. Copyright © 2019 Strtak et al. 2019 Strtak et al. This content is distributed under the terms of the Creative Commons Attribution 4.0 International license.

### NS1-2 viroporin activity maps to the putative viroporin domain.

**FIG 6:**
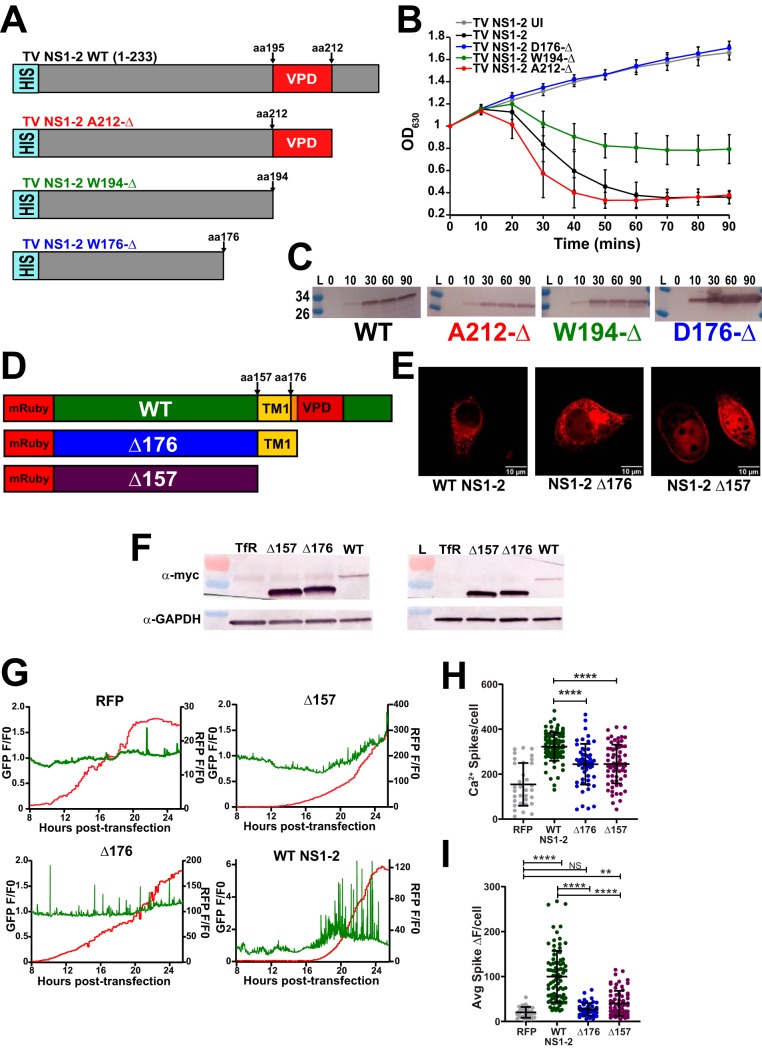
NS1-2 viroporin mutants do not increase cytoplasmic Ca^2+^. (A) Schematic of bacterially expressed TV NS1-2 C-terminal truncation mutants to functionally map the viroporin domain. (B) In the lysis assay, truncating the C-terminal domain to amino acid 212 (red) results in wild-type activity (black), but truncating to W194 (green) impairs activity. Truncating to D176 (blue) abrogates viroporin activity, suggesting that the viroporin domain functionally spans from aa177-212. UI, uninduced. (C) Western blots verifying protein expression in the lysis assay. (D) Schematic for the mammalian C-terminal truncation mutant constructs. (E) Immunofluorescence (IF) data for truncation mutants. The Δ157 mutant is cytoplasmic (far right), whereas the Δ176 mutant, which retains one transmembrane segment, is membrane localized (middle). (F) Western blot data confirm the Δ157 and Δ176 mutant constructs. The left blot is run with 20 μl/well to visualize wild-type (WT) NS1-2, whereas the right blot is run with 5 μl/well to resolve the size difference between the Δ157 and Δ176 NS1-2 mutants. TfR, transfection reagent. (G) Representative Ca^2+^ traces for WT NS1-2 and truncation mutants. (H) Both the Δ157 and Δ176 truncation mutants have significantly fewer Ca^2+^ spikes/cell compared to wild-type full-length RFP-NS1-2. (I) Compared to full-length RFP-NS1-2, both the Δ157 and Δ176 truncation mutants have significantly reduced Ca^2+^ spike amplitudes, resulting in a change in cytosolic fluorescence (Δ*F*) that phenotypically mimics RFP alone. Data shown are means ± SD from ≥8 fields of view. **, *P* < 0.01; ****, *P* < 0.0001; NS, not significant. *N* ≥ 3 for each experiment.

Our computational studies above identified a putative TV NS1-2 VPD from aa195-212. To determine whether the NS1-2 viroporin activity maps to this putative VPD, we generated C-terminal truncation mutants in bacterial expression vectors with deletions after aa212 (A212-Δ), after aa194 (W194-Δ), or after aa176 (D176-Δ) and characterized them in the lysis assay (Fig. 6A). We found that the A212-Δ truncation (red) had strong lysis activity comparable to full-length NS1-2 (black) (Fig. 6B). In contrast, the D176-Δ truncation (blue) exhibited no lysis activity, comparable to uninduced NS1-2 (gray) (Fig. 6B). Immunoblot analysis confirmed that protein expression correlated with viroporin activity and that the impaired activity of W194-Δ was not due to lower expression levels, since the expression was comparable to that of full-length protein and A212-Δ (Fig. 6C). Since the W194-Δ truncation (green) had impaired viroporin activity, this suggests that the VPD functionally extends to aa177-212.

Next, we characterized truncation mutants for their activation of aberrant Ca^2+^ signaling in MK2-G6s cells. Since recombinant expression of full-length RFP-NS1-2 induced aberrant Ca^2+^ signaling (Fig. 5D), we tested whether truncating the putative viroporin domain alone (Δ176) or both TMDs (Δ157) would compromise NS1-2-induced Ca^2+^ signaling (Fig. 6D). First, we examined the subcellular distributions and expression levels of the constructs. While the full-length and Δ176 truncation both appeared reticular, the Δ157 truncation had cytoplasmic distribution, consistent with it lacking both TMDs (Fig. 6E). Immunoblot analysis shows that the expression of both truncations was much greater than that of full-length NS1-2 (Fig. 6F, left blots), and by loading less lysate, we can better resolve the 2-kDa size difference in the Δ157 and Δ176 truncations (Fig. 6F, right blots). Next, we examined whether these truncations could induce Ca^2+^ signaling by long-term live-cell Ca^2+^ imaging in MK2-G6s cells. Individual cell traces illustrate that neither the Δ157 nor Δ176 truncation dramatically increased Ca^2+^ signaling similar to full-length RFP-NS1-2 (Fig. 6G). Quantitation of the Ca^2+^ spikes per cell showed that while both truncations exhibited higher Ca^2+^ signaling than RFP alone (Fig. 6H), the amplitude of these spikes was significantly reduced compared to full-length RFP-NS1-2 (Fig. 6I). The significant reduction in the number and amplitude of Ca^2+^ spikes/cell for both mutants highlights the critical importance of an intact VPD for disrupting host Ca^2+^ signaling. Together this work demonstrates that TV NS1-2 is an ER-targeted viroporin that induces aberrant Ca^2+^ signaling.

### Noroviruses exhibit aberrant Ca^2+^ signaling during infection and expression of NS1-2.

**FIG 7:**
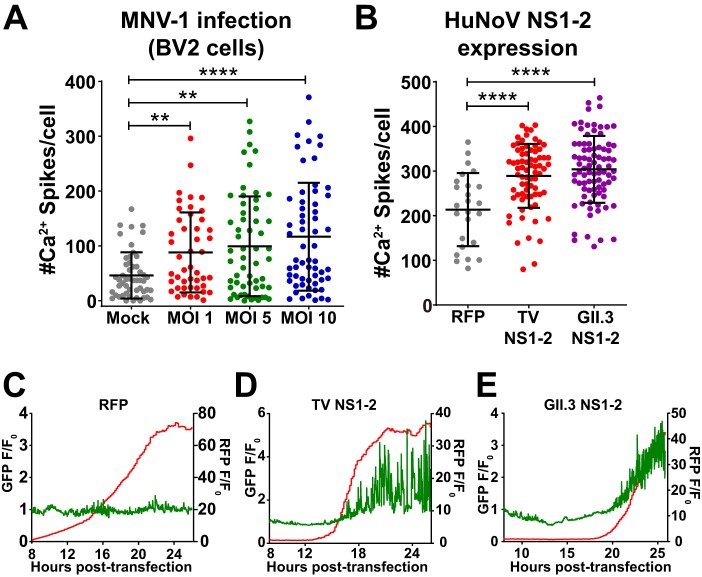
(A) Ca^2+^ spike analysis for MNV-1 CW-1 infection of GCaMP6s-expressing BV2 cells. Like TV, MNV infection causes aberrant Ca^2+^ signaling that increases in a dose-dependent manner. (B) Recombinant expression of GII.3 (U201) NS1-2 induces aberrant Ca^2+^ signaling, similar to TV NS1-2. (C) Representative Ca^2+^ trace data upon expression of RFP. (D) Representative Ca^2+^ trace shows that RFP-tagged TV NS1-2 increases the number and amplitude of Ca^2+^ spikes upon expression. (E) Representative Ca^2+^ trace for RFP-tagged GII.3 NS1-2 shows that GII.3 NS1-2 also increases the number and amplitude of Ca^2+^ spikes upon expression. Imaging experiments are quantitated based on ≥30 cells/condition. **, *P* < 0.01; ****, *P* < 0.0001. *N* ≥ 3 for all experiments.

Many aspects of HuNoV pathogenesis remain unknown, but elevation of cytosolic Ca^2+^ is implicated in many other enteric virus infections (31, 37, 38, 59, 66). The identification of aberrant Ca^2+^ signaling by TV and viroporin activity of NS1-2 could provide new insights into HuNoV pathogenesis if this activity is also evident in noroviruses. Thus, we wanted to know whether the aberrant Ca^2+^ signaling observed was specific to TV or shared among noroviruses. To test this, we infected GCaMP6s-expressing BV-2 cells with MNV-1 CW1 at an MOI of 1, 5, or 10 and performed long-term Ca^2+^ imaging, as described in Materials and Methods. Like TV infection, Ca^2+^ signaling in MNV-infected cells increases concomitant with infectious dose (Fig. 7A) and manifests as an increase in dynamic Ca^2+^ signaling (Movie S5). Interestingly, mock-inoculated BV2-GCaMP6s exhibited a greater number of Ca^2+^ spikes than mock-inoculated MK2-GCaMP6s cells, but this is likely due to differences in basal Ca^2+^ signaling between immune and epithelial cells (60–62).

10.1128/mSphere.00506-19.9 MOVIE S5 Representative time-lapse movie of BV2-GCaMP6s cells either mock inoculated (left) or infected with MNV-1 at an MOI of 5 (right). Infected cells show increased basal GCaMP6s fluorescence and an increased number of Ca^2+^ signaling events. Quantification of this imaging run can be seen in Fig. 7. Download Movie S5, MPG file, 19.6 MB. Copyright © 2019 Strtak et al. 2019 Strtak et al. This content is distributed under the terms of the Creative Commons Attribution 4.0 International license.

We next sought to determine whether the NS2 viroporin function we discovered in TV NS1-2 was conserved in the NS2 of any other calicivirus. The multiple-sequence alignment we performed for NS1-2 from other caliciviruses shows high variability in both the amino acid sequence and length of the NS1 region, but the C-terminal domain (CTD, NS2) of NS1-2 remains highly conserved (Fig. S1B). For MNV, NS1-2 confers infection persistence through the presence of a glutamic acid at position 94 and antagonizes the interferon pathway during infection, but this is almost exclusively through activity of NS1 (19, 63–65). Aside from subcellular localization experiments, very little is known about NS1-2 function during replication or infection in HuNoVs. To examine whether HuNoV NS1-2 activates aberrant Ca^2+^ signaling similar to TV NS1-2, we generated a mammalian expression vector of GII.3 NS1-2 N-terminally tagged with RFP and expressed it in MK2-G6s cells by transient transfection to perform Ca^2+^ imaging. Compared to the RFP control, expression of recombinant GII.3 NS1-2 causes aberrant Ca^2+^ signaling and significantly increases the number Ca^2+^ spikes similar to TV expression of RFP-NS1-2 (Fig. 7B). Representative Ca^2+^ traces from transfection show that GII.3 NS1-2-mediated Ca^2+^ signaling is dynamic and occurs at the onset of protein expression (Fig. 7E and Movie S6). Thus, these data show that noroviruses cause aberrant Ca^2+^ signaling during infection and disrupt host Ca^2+^ signaling through production and expression of the nonstructural protein NS1-2.

10.1128/mSphere.00506-19.10 MOVIE S6 Representative signaling from viroporin transfection experiments in LLC-MK2 GCaMP6s cells. The movies show that cells exhibit aberrant Ca^2+^ signaling upon expression of RFP-tagged TV NS1-2 (top) or GII.3 HuNoV NS1-2 (bottom). Quantification of this imaging run can be seen in Fig. 7. Download Movie S6, MPG file, 4.7 MB. Copyright © 2019 Strtak et al. 2019 Strtak et al. This content is distributed under the terms of the Creative Commons Attribution 4.0 International license.

## DISCUSSION

As obligate intracellular pathogens, viruses are adept at exploiting host pathways to facilitate replication. Viruses from many different taxonomic families activate aberrant Ca^2+^ signaling because Ca^2+^ signals are used by all cells to regulate a vast array of cellular functions. Therefore, this represents a powerful strategy to reconfigure host cell physiology via targeted disruption of host Ca^2+^ homeostasis. The overarching goal of this study was to determine whether dysregulation of Ca^2+^ signaling is a characteristic of caliciviruses and whether this is due to the production of a viroporin protein similar to picornaviruses. To address these questions, we studied TV, as a model calicivirus, using a combination of live-cell Ca^2+^ imaging and other classical techniques. The major new findings of this study are as follows. (i) TV infection causes aberrant Ca^2+^ signaling that coincides with viral protein synthesis and replication. (ii) Cellular Ca^2+^ is critical for TV replication, and buffering of cytosolic Ca^2+^ severely reduced viral yield. (iii) TV NS1-2 has viroporin activity and dysregulates Ca^2+^ signaling in mammalian cells similar to TV infection. (iv) NS1-2 viroporin activity maps to a C-terminal integral membrane viroporin domain, and truncation of this domain abrogates the NS1-2-induced activation of Ca^2+^ signaling. Finally, we extended these observations to show that both MNV-1 infection and expression of recombinant HuNoV NS1-2 induces aberrant Ca^2+^ signaling. To our knowledge, these results are the first to show exploitation of Ca^2+^ signaling by a calicivirus and identification of NS1-2 as a Ca^2+^-disrupting viroporin. These findings further extend the functional homology between the calicivirus nonstructural proteins and their picornavirus positional homologs.

The exploitation of host Ca^2+^ signaling to facilitate virus replication is a common feature of many viruses (31). Our finding that TV coopts Ca^2+^ signaling is consistent with previous studies showing that elevated Ca^2+^ levels are important for picornavirus replication, especially since caliciviruses and picornaviruses utilize a similar replication strategy (44). Similar to other Ca^2+^-disrupting viruses, TV also induces aberrant Ca^2+^ signaling peak of virus replication, many hours after cell entry. This is consistent with the reduced virus yield in media with reduced extracellular Ca^2+^ or treatments to buffer cytosolic Ca^2+^ (BAPTA-AM) or block refilling of ER Ca^2+^ stores (TG). Further, as we recently reported for RV infection, the TV-induced increase in cytosolic Ca^2+^ manifests as many discrete Ca^2+^ signals rather than a monophasic increase in Ca^2+^ over the infection (66). This raises the following questions. (i) What cellular pathways are activated by this Ca^2+^ signaling? (ii) How do they benefit TV replication? Both RV and EV have been shown to exploit Ca^2+^ signaling to activate the biosynthetic early stages of autophagy, which facilitates virus replication through rearrangement of cellular membranes to form replication complexes (67). MNV infection of primary macrophages or the RAW264.7 cell line activates autophagy, but in contrast to RV and EV, autophagy limits MNV replication (68). Thus, it remains to be determined whether autophagy plays a role in calicivirus replication complex assembly or whether Ca^2+^ signaling regulates autophagy activation during calicivirus infection. Further, elevated Ca^2+^ signaling may serve to modulate cellular apoptotic responses. Strong monophasic increases in cytosolic Ca^2+^ activate apoptosis through mitochondrial Ca^2+^ overload, but transient and oscillatory Ca^2+^ fluxes serve as prosurvival signals (69). Activation of apoptosis has been seen in norovirus- and feline calicivirus-infected cells, and caspase activation is critical for cleavage and release of MNV NS1 from NS1-2, which in turn modulates cellular innate immune responses (19, 22). Additionally, previous work with MNV-1 CR6 shows that NS1-2 from this norovirus is cleaved by caspase-3 during late infection. Apoptotic induction coincided with viral egress, suggesting that activation of apoptosis and cleavage of NS1-2 by caspase-3 occur to facilitate viral spread after viral replication and virion assembly (17). Thus, increased transient Ca^2+^ signaling may serve to counteract apoptosis activation until necessary to help prolong cell viability and maximize virus replication.

Within the superfamily of picornavirus-like positive-sense RNA viruses, there is positional homology between the ORF1 nonstructural proteins of caliciviruses (and likely astroviruses) and the P2-P3 nonstructural proteins of picornaviruses (24, 46, 47). We used this framework to determine whether TV NS1-2 exhibited viroporin activity, since the positional homolog, the picornavirus 2B protein, is a well-established Ca^2+^-conducting viroporin (35, 45). We found that TV NS1-2 has viroporin activity, similar to 2B and RV NSP4, and the viroporin activity mapped to the integral membrane NS2 domains. Since both the N and C termini are likely oriented in the cytoplasm, NS1-2 is classified as a class IIB viroporin, similar to the picornavirus 2B proteins (35, 45). This topology is supported by the cytosolic accessibility of the NS1 domain and the need for the C terminus to also be localized in the cytosol to enable cleavage by the NS6 protease. Further, the similarity of Ca^2+^ signaling induced by TV and HuNoV RFP-NS1-2 raises the question of whether NS1-2 viroporin activity is conserved throughout the *Caliciviridae* family. Though 2B and NS1-2 lack appreciable primary sequence homology, this is not surprising because viroporins, even from the same virus family, often share only the common viroporin motifs (i.e., [i] having an amphipathic α-helix, [ii] having a cluster of basic residues, and [iii] being oligomeric) (34, 39, 40). We found that among NS1-2 from different caliciviruses, these characteristic features are conserved, so we predict that viroporin activity of NS1-2 is a common function. Furthermore, since blunting cytosolic Ca^2+^ signaling with BAPTA-AM reduced TV replication, blocking NS1-2 viroporin activity with mutations or drugs should also reduce replication. This is supported by a previous study showing that recombinant coxsackie B3 virus with mutations of the 2B viroporin exhibited significantly impaired replication or was completely replication deficient (70). Analogous studies can be done using the TV reverse genetics system once residues critical for viroporin activity are identified through mutagenesis screens of the TV NS1-2 viroporin domain we mapped in this study.

The increased Ca^2+^ signaling observed in TV-infected cells is phenotypically similar to that induced by recombinant expression of full-length NS1-2, but the Ca^2+^ signaling is abrogated by truncation of the viroporin domain. Further, NS1-2 primarily localized to the ER, which is a major intracellular Ca^2+^ storage organelle. Thus, our model predicts that NS1-2 directly releases Ca^2+^ from the ER; however, it is likely that both NS1-2 and activation of host Ca^2+^ signaling pathways contribute to the observed Ca^2+^ signals. Ca^2+^ signals from NS1-2 require it to directly conduct Ca^2+^ and have a high enough conductance that the ER Ca^2+^ release event can be detected by a fluorescent Ca^2+^ indicator, yet these unitary events are challenging to detect even for large channels like the inositol trisphosphate receptor (IP3R) (71). Future studies using patch clamp electrophysiology are needed to confirm that NS1-2 conducts Ca^2+^ and determine its conductivity. Nevertheless, based on the similarities between NS1-2 and other Ca^2+^-conducting viroporins, EV 2B and RV NSP4, NS1-2 viroporin activity would reduce ER Ca^2+^ levels, and this in turn will activate host Ca^2+^ signaling pathways. First, the moderately increased steady-state cytosolic Ca^2+^ levels could foster more ER Ca^2+^ release by potentiating the IP3R Ca^2+^ release channel (72). Second, reduced ER Ca^2+^ levels activate the store-operated Ca^2+^ entry (SOCE) pathway, wherein decreased ER Ca^2+^ levels activate the ER Ca^2+^ sensing protein stromal interaction molecule 1 (STIM1). Activated STIM1 translocates to ER microdomains adjacent to the plasma membrane and opens Ca^2+^ influx channels, like Orai1, to elevate cytosolic Ca^2+^ (32, 33). This Ca^2+^ influx, in concert with SERCA, helps to refill ER stores for continued signaling.

HuNoV and human sapoviruses cause outbreaks of acute gastroenteritis (AGE) and are a major cause of foodborne illnesses. However, the molecular mechanisms of how these caliciviruses cause vomiting and diarrhea, the chief symptoms of AGE, have not been characterized. The dysregulation of Ca^2+^ signaling by TV may provide insights into the pathophysiology of enteric caliciviruses. Both IP_3_-mediated ER Ca^2+^ release and SOCE have been shown to activate chloride secretion from epithelial cells (73, 74). In studies of other viroporins, the viroporin-induced elevated cytosolic Ca^2+^ induces cytoskeleton rearrangement, leading to disassembly of tight junctions and loss of barrier integrity (40). Hyperactivation of chloride secretion and loss of tight junctions would contribute to excess fluid secretion and diarrhea. In our study, we have shown that dysregulated Ca^2+^ signaling is a feature of calicivirus infection using TV. Additionally, our data with recombinant GII.3 NS1-2 shows aberrant Ca^2+^ signaling at the onset of expression similar to what we observe with recombinant expression of TV NS1-2 (Fig. 7). This suggests that HuNoV NS1-2 may be functioning as a viroporin, similar to TV NS1-2. Thus, future studies can further examine the role of aberrant Ca^2+^ signaling in calicivirus pathophysiology using human intestinal enteroid cultures that support the replication of many HuNoV strains (4).

In summary, we have shown that TV activates aberrant Ca^2+^ singling during infection, and cellular Ca^2+^ is critical for robust TV replication. Further, we found that the NS2 domain of the NS1-2 nonstructural protein is a viroporin that alone induces Ca^2+^ signaling similar to TV infection. Together, these results indicate that NS1-2 is functionally analogous to EV 2B and RV NSP4. While little is known about the function(s) of NS1-2, and particularly the NS2 domain of NS1-2, the similarity with other Ca^2+^-conducting viroporins may provide a broader insight for understanding NS1-2 functions. Finally, antiviral drugs against viroporins have been developed for influenza virus M2 and HIV Vpu (35). Thus, the NS1-2 viroporin may be a viable antiviral drug target against caliciviruses.

## MATERIALS AND METHODS

### Cell lines, GECI lentiviruses, and viruses.

All experiments were performed in LLC-MK2 cells. Lentivirus packaging and recombinant protein expression for Western blot lysate production was performed in HEK293FT cells (ATCC CRL-3216). Cell lines were grown in high-glucose Dulbecco modified Eagle medium (DMEM) (catalog no. D6429; Sigma) containing 10% fetal bovine serum (FBS) (Corning lot no. 35010167) and antibiotic/antimycotic (Invitrogen), and maintained at 37°C with 5% CO_2_. Lentivirus packaging in HEK293FT cells was performed as previously described (42). Briefly, LLC-MK2 cells were transduced with a lentivirus vector encoding GCaMP6s 1 day after seeding (∼85% confluence). We confirmed positive expression of GCaMP6s 48 to 72 h after transduction and then passaged cells 1:2 and added hygromycin (100 μg/ml) for selection of the LLC-MK2-GCaMP6s cell lines, henceforth referred to as MK2-G6s. We determined GCaMP6s activity and dynamic range using thapsigargin (TG) (0.5 μM). Tulane virus (TV) stocks were made in-house by infecting cells with an MOI of 0.01 and harvesting at ∼95% cytopathic effect (CPE). Virus titer was determined by plaque assay. Irradiated virus controls were made by gamma-irradiating TV stocks for 19 h. MNV-1 CW1 virus was a kind gift from Herbert Virgin, and BV2 cells were a kind gift from Christiane Wobus. MNV-1 stocks were made by infecting BV2 cells at an MOI of 0.01 and harvesting at ∼95% CPE. Virus titers were determined by plaque assay on BV2 cells using a similar protocol as for TV plaque assays except the final overlay was 1.2% Avicel.

### Replication assays.

LLC-MK2 cells were seeded at 125,000 cells/well in 24-well plates (Costar 3524; Corning) and inoculated the next day with TV at an MOI of 1 for 1 h. Inoculum was removed, and cell medium was replaced containing different extracellular Ca^2+^ conditions (0 mM Ca^2+^, 4 mM Ca^2+^), intracellular Ca^2+^ chelator 50 μM 1,2-bis(2-aminophenoxy)ethane-*N*,*N*,*N*',*N*'-tetraacetic acid-acetoxymethyl ester (BAPTA-AM), or the sarco/endoplasmic reticulum calcium ATPase (SERCA) blocker thapsigargin (TG). Ca^2+^-free DMEM was purchased from Gibco (catalog no. 21068-028). Standard high-glucose DMEM (Sigma) has 1.8 mM CaCl_2_, which we refer to as “2 mM Ca^2+^,” and media with 4 mM Ca^2+^ was made by adding 2 mM CaCl_2_ to the standard high-glucose DMEM (Sigma). We maintained TV-infected cells under these conditions until the positive control (normal media) had ∼90% CPE. Progeny virus was harvested by three freeze/thaw cycles, and the virus yield was determined by plaque assay. For plaque assays, cells are seeded at 75,000 cells/well in 24-well plates, and 2 days after seeding, the cells were inoculated for 1 h with 10-fold serial dilutions of the sample. Then, we removed the inoculum and added the overlay. Overlays for plaque assays were made by mixing equal parts of 1.2% Avicel (FMC Corporation) and 2× DMEM (Gibco). Plaque assays were harvested at 72 h and fixed and stained with crystal violet (3% solution) to visualize plaques. Titer is represented as plaque-forming units per milliliter (PFU/ml). To compare plaque size, images of wells were analyzed using Nikon Elements software to measure the longest diameter, and the resulting data were graphed using GraphPad Prism software.

### One-step growth curves.

One-step growth curves for TV were performed using a modified protocol from previous reports (11, 15). Briefly, LLC-MK2 cells were inoculated with TV at an MOI of 1 in serum-free DMEM (0% FBS DMEM). At 1 h postinfection (HPI), the inoculum was removed and replaced with 0% FBS DMEM. Cells were harvested at 0, 4, 6, 8, 10, 12, 16, 20, 24, and 28 HPI, and virus yield was determined by plaque assay. Each biological replicate was performed in duplicate.

### Long-term Ca^2+^ imaging experiments.

Calcium imaging experiments were set up by adapting a protocol detailed in previous reports (66). For TV infections, MK2-G6s cells were seeded at 78,500 cells/well in 15 μ-slide 8-well chambers (Ibidi, Germany) and infected the next day with TV at the indicated MOI. After 1 h, the inoculum was removed and replaced with FluoroBrite DMEM (Gibco). For studies involving pharmacological compounds, the FluoroBrite DMEM was mixed with dimethyl sulfoxide (DMSO) (0.1%; vehicle control) or the indicated pharmacological compounds dissolved in DMSO. For MNV-1 infections, BV2-G6s cells were seeded at 150,000 cells/well in 15 μ-slide 8-well chambers and infected the next day with MNV-1 strain CW1 at the indicated MOI. After 1 h, the inoculum was removed and replaced with FluoroBrite DMEM as described above. Then the slide was mounted into a stage-top environmental chamber (Okolab H301-Mini) maintained at 37°C with humidity control and 5% CO_2_. Time-lapse live-cell Ca^2+^ imaging experiments were conducted from ∼2 HPI until ∼18 to 24 HPI on a Nikon TiE epifluorescence microscope using a Spectrax LED light source (Lumencor) and a 20× Plan Apo (numerical aperture, 0.75) objective. Images were acquired at 1 or 2 images/well point/minute. Images were acquired and analyzed using the NIS elements advanced research software package (Nikon). Prior to image analysis, background camera noise was subtracted from the images using an averaged file of 10 no-light camera images. Cells that underwent division during the imaging run were excluded from analysis. Intracellular Ca^2+^ signaling over time was quantified by calculating the number of Ca^2+^ spikes per cell. This was determined as follows: raw fluorescence intensity values were measured from individual cells using Nikon software, then exported to Microsoft Excel to normalize the fluorescence to the first image (*F*/*F*_0_). The Ca^2+^ spikes were calculated by subtracting each normalized fluorescence measurement from the previous measurement to determine the change in GCaMP6s fluorescence (Δ*F*) between each time point. Ca^2+^ signals with a Δ*F* magnitude of >5% were counted as Ca^2+^ spikes. For each condition tested, Ca^2+^ spikes in ≥30 cells were determined.

### Heatmap generation.

To generate heatmaps of the normalized GCaMP6s fluorescence over time for long-term Ca^2+^ imaging experiments, we used the TidyR (75) and ggplot2 (76) packages available through R studio. Normalized GCaMP6s data from Excel was used to create an R-compatible file (.csv) containing the normalized fluorescence and the acquisition time data for the data set, and the file was imported into R. We used the TidyR package to organize data into a format accessible by ggplot2. We then used ggplot2 to generate heatmaps.

### Prediction of viroporin motifs *in silico*.

We used the Hydropathy Analysis program at the Transporter Classification Database to generate Kyte and Doolittle Hydropathy and Amphipathic moment plots to identify putative viroporin motifs within full-length TV NS1-2 (77). Secondary structure, membrane topology, and membrane integration predictions were performed using PSIPred prediction analysis suite (website http://bioinf.cs.ucl.ac.uk/introduction/) (55). Helical wheel plots to identify clustered basic residues within the putative viroporin domain were generated using the PepWheel analysis program at Heliquest (website http://heliquest.ipmc.cnrs.fr/) (56).

### Expression vectors.

E. coli expression constructs for the lysis assay were generated via ligation-independent cloning (LIC) using the pET46-Ek/LIC kit (MilliporeSigma, Darmstadt, Germany). The pET46-Ek/LIC constructs all have an N-terminal six-histidine tag. Mammalian expression vectors were generated by inserting c-myc tag and mRuby3 red fluorescent protein (RFP) upstream of full-length NS1-2 and then subcloning this into the pTagRFP-N vector in place of TagRFP (Epoch Life Sciences, Missouri City, TX). This construct is referred to as RFP-NS1-2. The NS1-2(Δ176) and NS1-2(Δ157) truncation mutations in both bacterial and mammalian expression vectors were generated using the NEB Q5 site-directed mutagenesis kit (New England Biolabs, Ipswich, MA). Primer sequences used for the bacterial and mammalian expression vectors are listed in Fig. S4 in the supplemental material. The sequences of all constructs were verified using universal primers specific to the construct backbone (GENEWIZ, South Plainfield, NJ). The mammalian expression vector for EV 2B was generated by cloning the 2B from enterovirus 71 upstream into pTagRFP-N, and the construction of the NSP4-TagRFP expression vector was previously described (78).

### Transfection experiments.

MK2-G6s cells were seeded in 15 μ-slide 8-well chambers (Ibidi, Germany) and at 85% confluence transfected with mammalian expression constructs in Opti-MEM (ThermoFisher) and Lipofectamine 2000 (Invitrogen). Transfection was optimized so cells received 400 ng of plasmid DNA and 0.5 μl of Lipofectamine 2000 per well. Trichostatin A (TSA) (10 μM) was added from 1 to 3 h posttransfection. TSA is a histone deacetylase (HDAC) inhibitor used to increase expression from the vectors (79–81). Time-lapse Ca^2+^ imaging was performed beginning 8 h posttransfection to capture expression kinetics and up to 24 h posttransfection to measure changes in Ca^2+^ signaling during expression of the RFP-tagged proteins.

### Deconvolution microscopy.

LLC-MK2 cells were seeded in 15 μ-slide 8-well chambers (Ibidi, Germany) and transfected 1 day prior to imaging. Cells were transfected with intracellular markers for the plasma membrane (LCK-GFP; Addgene plasmid #61099), endoplasmic reticulum (pLV-ER GFP; Addgene plasmid #80069), Golgi apparatus (pLV-Golgi GFP; Addgene plasmid #79809), and mitochondria (HyPer-dMito; Evrogen). Control wells received TagRFP (Evrogen), while experimental wells received either full-length RFP-NS1-2, RFP-NS1-2(Δ157), or RFP-NS1-2(Δ176). Cells were imaged 24 h posttransfection on the DeltaVision LIVE high-resolution deconvolution microscope (GE Healthcare) using the 60×/1.4 Plan-Apo NA oil objective (Olympus), and acquired using a pco.edge sCMOS_5.5 camera. Images were acquired and deconvolved in SoftWoRx software. After the images were deconvolved, they were further processed in FIJI (ImageJ) to adjust for brightness/contrast and pseudocoloring (82).

### E. coli lysis assay.

E. coli lysis assays were performed as previously described (42). Briefly, pET46-Ek/LIC constructs of the full-length TV NS1-2 and truncation mutants were transformed into E. coli BL21(DE3)pLysS cells. Transformations were plated on LB containing 1% glucose, 100 μg/ml ampicillin, and 35 μg/ml chloramphenicol and grown at 37°C overnight. Isolated colonies were picked the next day and cultured overnight in liquid LB containing 1% glucose, 100 μg/ml ampicillin, and 35 μg/ml chloramphenicol at 37°C in an orbital shaker at 250 rpm. The next day, overnight cultures were subcultured by 1:100 dilution into 200 ml LB containing 1% glucose, 100 μg/ml ampicillin, and 35 μg/ml chloramphenicol. Subcultures were grown at 37°C in an orbital shaker at 250 rpm for ∼3 h to an optical density at 630 nm (OD_630_) between 0.3 and 0.5 and then induced with 1 mM isopropyl-β-d-thiogalactopyranoside (IPTG). Absorbance measurements at 630 nm (OD_630_) were taken every 10 min for 90 min and normalized to the induction OD_630_ to determine the percent growth or lysis over time after induction. Each experiment was performed ≥3 times. Protein expression was determined by SDS-PAGE using a 4 to 20% Tris-glycine gel (Bio-Rad, Hercules, CA) and Western blotting for the six-histidine tag. An uninduced culture served as the negative control for viroporin activity and NS1-2 synthesis.

### Membrane association experiment.

Membrane association experiments were performed using a modified protocol from previously reported experiments (37, 42). For bacterial membrane association, we collected lysed membranes from a 200-ml induced culture. For mammalian membrane association experiments, we collected lysed membranes in 500 μl of radioimmunoprecipitation assay (RIPA) buffer with protease inhibitor from a transfected well of a six-well plate. Lysed membranes were centrifuged at 21,000 × *g* for 20 min, and supernatants were decanted. Pellets were resuspended in phosphate-buffered saline (PBS) and sonicated three times for 1 min on ice. Total lysate was collected after sonication. The membranes were then pelleted by ultracentrifugation at 49,000 × *g* for 1 h using a TLA-100.3 rotor in an Optima TL ultracentrifuge (Beckman Coulter, Indianapolis, IN), and the supernatant was collected for the soluble fraction. Finally, the membrane fraction pellet was resuspended in PBS containing 1% SDS to solubilize membrane proteins. Samples from the total lysate, soluble fraction, and membrane fractions were analyzed by Western blotting.

### Production of TV and Vpg antisera.

For the anti-TV antisera to detect VP1, adult male and female CD-1 mice (purchased from the Center for Comparative Medicine, Baylor College of Medicine) were immunized five times with CsCl_2_ gradient-purified TV at 10 μg/dose in AddaVax adjuvant (InvivoGen). Immunizations were given at 3-week intervals. For the anti-Vpg antisera, adult BALB/c were immunized three times with 10 to 20 μg of purified Vpg expressed in E. coli per dose. The priming dose was given in Freund’s complete adjuvant, and the subsequent boosts were given in Freund’s incomplete adjuvant. Figure S4 shows immunoblot analysis of the antisera. All experiments were performed in accordance with the recommendations in the *Guide for the Care and Use of Laboratory Animals* (83).

### Immunoblot analysis.

Samples were prepared using procedures adapted from reference 37. Briefly, samples were mixed with 5× sample buffer containing 2-mercaptonethanol and boiled for 10 min at 100°C. Samples were then run on a 4 to 20% Tris-glycine gel (Bio-Rad, Hercules CA) and transferred onto a nitrocellulose membrane using the Transblot Turbo transfer system (Bio-Rad, Hercules, CA). To detect the bacterial constructs of NS1-2 and NSP4, we used the mouse anti-His tag monoclonal antibody at 1:1,000 (Genscript, Piscataway, NJ). To detect mammalian expression constructs of NS1-2, we used the mouse anti-c-Myc monoclonal antibody (clone 9E10) at 1:1,000 (R&D Systems, MN). To detect TV structural protein VP1, we used the mouse anti-TV polyclonal antibody we made in-house by hyperimmunizing CD1 mice with purified TV particles. To detect TV nonstructural protein Vpg, we used the mouse anti-Vpg polyclonal antibody made by hyperimmunizing mice with bacterially expressed and purified Vpg. For loading control of mammalian cell lysates, we used the mouse anti-glyceraldehyde-3-phosphate dehydrogenase (anti-GAPDH) at 1:3,000 (Novus Biologicals, CO). For secondary detection of all primary antibodies used in these experiments, we used alkaline phosphatase-conjugated goat anti-mouse IgG at 1:2,000 (Southern Biotech, Birmingham, AL) and visualized using alkaline phosphatase substrate (Tris-base, nitro blue tetrazolium [NBT], 5-bromo-4-chloro-3-indolyl phosphate [BCIP]). We used a PageRuler 10- to 180-kDa prestained protein ladder for all of our Western blots (ThermoFisher).

### Statistical analysis.

Statistical analyses were completed using GraphPad Prism (version 7.03). Data in this article are presented as means ± standard deviations. Unless otherwise noted, all experiments in this article were performed in biological triplicate, with at least two technical duplicates per biological replicate, when applicable. We performed column statistics to collect descriptive statistics and to determine the normality of the data sets. We then used the unpaired Student’s *t* test for data sets with a parametric distribution or a Mann-Whitney test for data sets with a nonparametric distribution. Differences were determined statistically significant if the *P* value was <0.05. Authors had access to the data for this article, and all authors approved the final article.

### Data availability.

RConsole code for the heatmaps generated in this paper is available upon request.
