# Supplementary figures and images for: Recovirus NS1-2 Has Viroporin Activity That Induces Aberrant Cellular Calcium Signaling To Facilitate Virus Replication
Source: mSphere. 2019 Sep 18;4(5):e00506-19. doi: 10.1128/mSphere.00506-19 (PMC6751491; doi:10.1128/mSphere.00506-19)

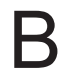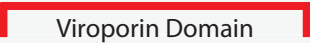

Supplement: FIG S1 [file mSphere.00506-19-sf001.pdf]

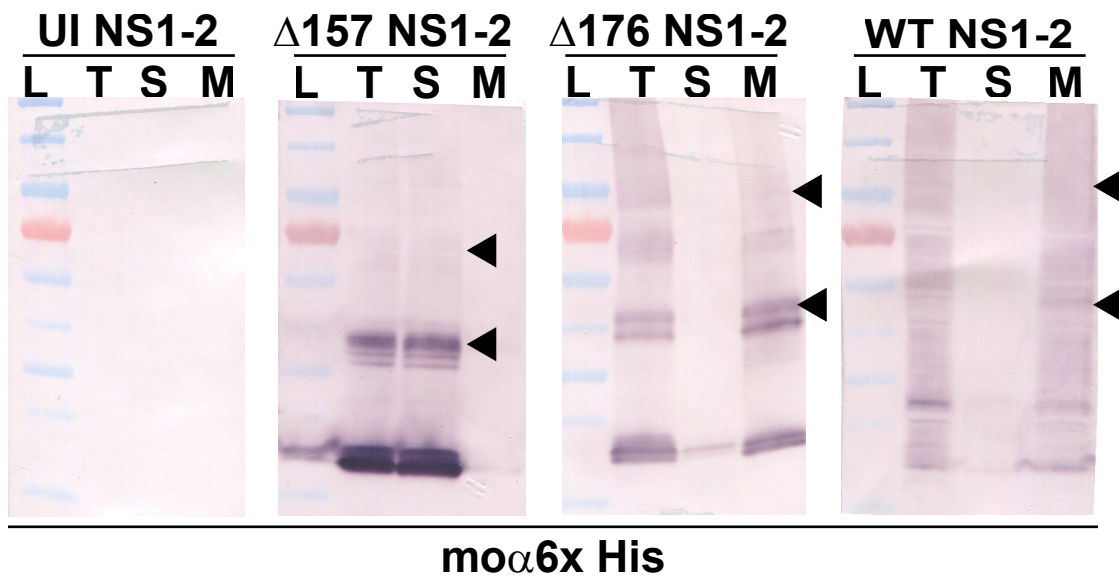

Supplement: FIG S3 [file mSphere.00506-19-sf003.pdf]
