# Supplementary material for: Recovirus NS1-2 Has Viroporin Activity That Induces Aberrant Cellular Calcium Signaling To Facilitate Virus Replication
Source: mSphere. 2019 Sep 18;4(5):e00506-19. doi: 10.1128/mSphere.00506-19 (PMC6751491; doi:10.1128/mSphere.00506-19)
Supplement: FIG S2 [file mSphere.00506-19-sf002.pdf]

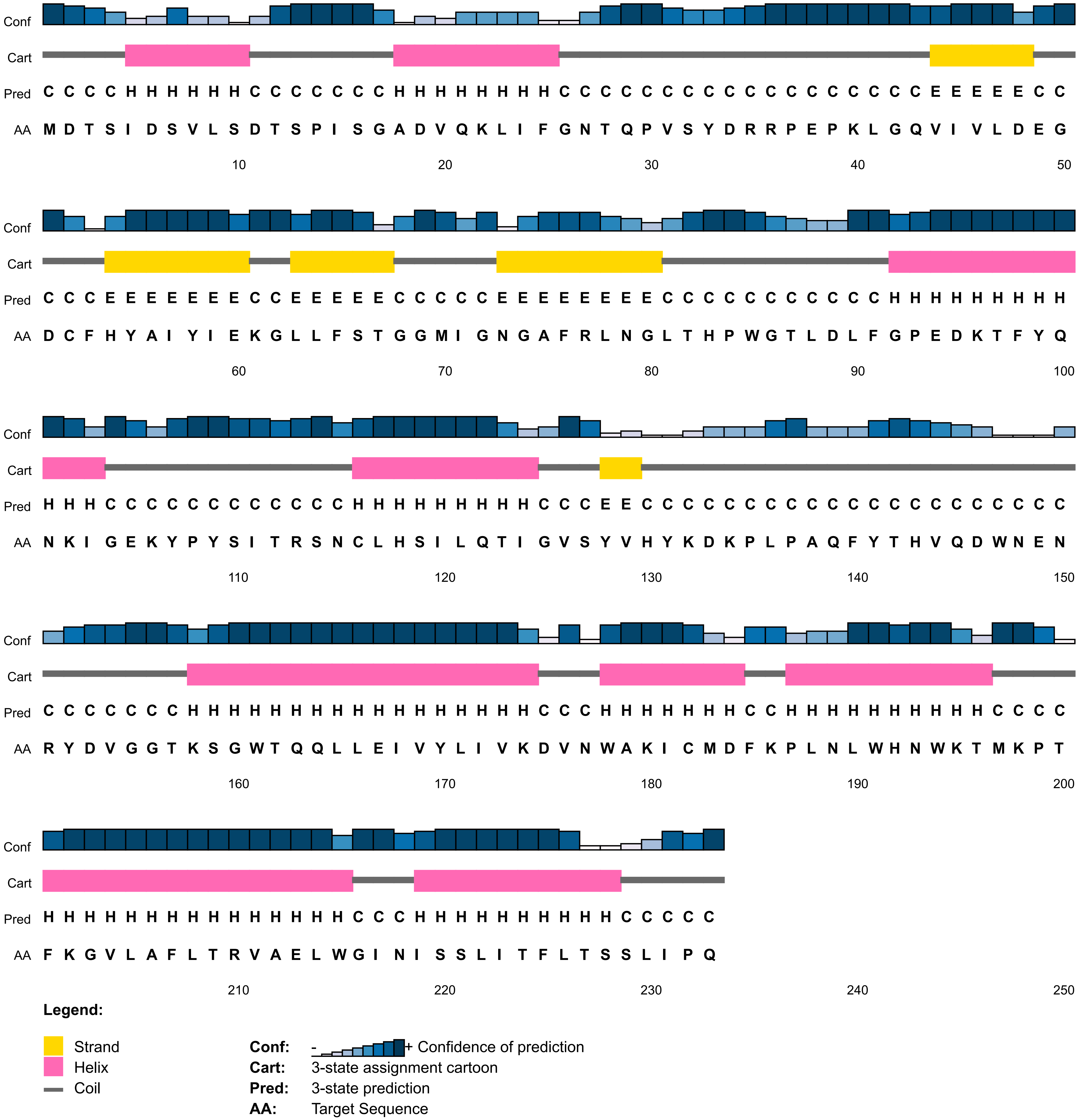
Conf
Cart
Pred C C C C H H H H H H C C C C C C C H H H H H H H H C C C C C C C C C C C C C C C C C C E E E E E C C
AA M D T S I D S V L S D T S P I S G A D V Q K L I F G N T Q P V S Y D R R P E P K L G Q V I V L D E G
10 20 30 40 50
Conf
Cart
Pred C C C E E E E E E E C C E E E E E C C C C C E E E E E E E E C C C C C C C C C C C H H H H H H H H H
AA D C F H Y A I Y I E K G L L F S T G G M I G N G A F R L N G L T H P W G T L D L F G P E D K T F Y Q
60 70 80 90 100
Conf
Cart
Pred H H H C C C C C C C C C C C C H H H H H H H H H C C C E E C C C C C C C C C C C C C C C C C C C C C
AA N K I G E K Y P Y S I T R S N C L H S I L Q T I G V S Y V H Y K D K P L P A Q F Y T H V Q D W N E N
110 120 130 140 150
Conf
Cart
Pred C C C C C C C H H H H H H H H H H H H H H H H H C C C H H H H H H H C C H H H H H H H H H H C C C C
AA R Y D V G G T K S G W T Q Q L L E I V Y L I V K D V N W A K I C M D F K P L N L W H N W K T M K P T
160 170 180 190 200
Conf
Cart
Pred H H H H H H H H H H H H H H H C C C H H H H H H H H H H C C C C C
AA F K G V L A F L T R V A E L W G I N I S S L I T F L T S S L I P Q
210 220 230 240 250
Legend:
Strand
Helix
Coil
Conf: - + Confidence of prediction
Cart: 3-state assignment cartoon
Pred: 3-state prediction
AA: Target Sequence
