# Supplementary material for: Recovirus NS1-2 Has Viroporin Activity That Induces Aberrant Cellular Calcium Signaling To Facilitate Virus Replication
Source: mSphere. 2019 Sep 18;4(5):e00506-19. doi: 10.1128/mSphere.00506-19 (PMC6751491; doi:10.1128/mSphere.00506-19)
Supplement: FIG S4 [file mSphere.00506-19-sf004.pdf]

A

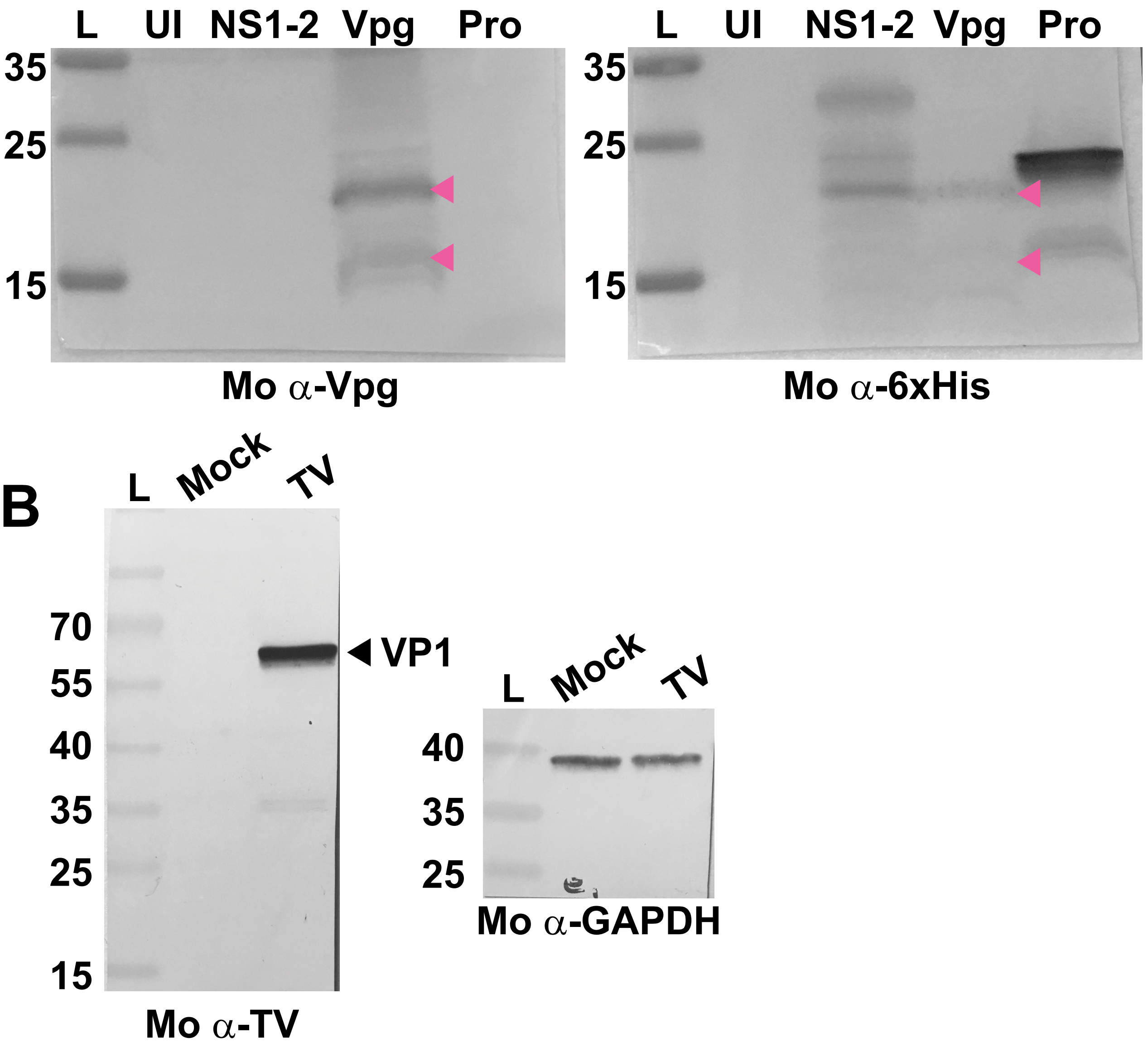


C

| Primer name | Sequence (5' to 3') |
|---|---|
| pET-46 EK/LIC NS1-2 Fwd | GACGACGACAAGATGGATACGTCCATAGATTCTGTGTTATCTGACACCAGC |
| pET-46 EK/LIC NS1-2 Rev | GAGGAGAAGCCCGGTTACTGTGGGATCAGTGATGAGGTAAGAAATGTGATCAAACTGG |
| pET-46 EK/LIC NS1-2 D176 Rev | GACGACAAGCCCGGTTAATCTTTGACAATAAGGTACACGATCTCGAG |
| pET-46 EK/LIC NS1-2 W194 Rev | GAGGAGAAGCCCGGTTACCAATTGTGCCAAAGGTTC |
| pET-46 EK/LIC NS1-2 A212 Rev | GAGGAGAAGCCCGGTTAAGCAACCCTTGTCAGGAATG |
| pET-46 EK/LIC NS1-2 Truncations Fwd | GACGACGACAAGATGGATACGTCCATAGATTCTGTGTTATC |
| pET-46 EK/LIC NS1-2 Δ157 Rev | GAGGAGAAGCCCGGTTTATTTGGTCCCTCCGACG |
| pET-46 EK/LIC NS1-2 Δ176 Rev | GAGGAGAAGCCCGGTTTAATTGACATCTTTGACAATGAGG |
| pTagRFP-N mRuby3-NS1-2 Δ157 Q5 Mut. Fwd | CGGAGGGACCTAAAGCGGATGGAC |
| pTagRFP-N mRuby3-NS1-2 Δ157 Q5 Mut. Rev | GTCGTACCTATTTTCATTCCAATCTTGGACATGTGTG |
| pTagRFP-N mRuby3-NS1-2 Δ176 Q5 Mut. Fwd | CAAAGATGTCTAATGGGCAAAGATTTG |
| pTagRFP-N mRuby3-NS1-2 Δ176 Q5 Mut. Rev | ACAATGAGGTACACGATCTCGAG |
